# Alveolar epithelial-like cell differentiation in a dynamic bioreactor: a promising 3D-approach for the high-throughput generation of lung cell types from human induced pluripotent stem cells

**DOI:** 10.1007/s44164-023-00052-1

**Published:** 2023-06-29

**Authors:** Michelle Müller, Yvonne Kohl, Anja Germann, Sylvia Wagner, Heiko Zimmermann, Hagen von Briesen

**Affiliations:** 1 Fraunhofer Institute for Biomedical Engineering IBMT, Sulzbach, Germany; 2 Molecular and Cellular Biotechnology/Nanotechnology, Saarland University, Saarbrücken, Germany; 3 Facultad de Ciencias del Mar, Universidad Cato´ Lica del Norte, Coquimbo, Chile

**Keywords:** Suspension bioreactor, SARS-CoV-2 pseudotypes, In vitro lung models, Spheroids

## Abstract

**Purpose:**

Human induced pluripotent stem cell (hiPSC)-derived lung cell types such as alveolar epithelial cells are promising for toxicological and pharmaceutical in vitro screenings. Reproducible differentiation processes are highly demanded, but protocols which are suitable for the high-throughput generation of lung cell types from hiPSCs are lacking.

**Methods:**

In this study, a new approach for the hiPSC-differentiation in alveolar epithelial-like cells type 2 under dynamic 3D-conditions in a suspension bioreactor is presented. Gene and protein expression analyses of key markers during the embryonal lung development have been performed in comparison to cells differentiated under static 2D-conditions to evaluate the differentiation efficacy of the new bioreactor-based approach. Finally, the resulting cells were infected by SARS-CoV-2 pseudotypes to demonstrate their functionality and suitability for e.g. COVID-19 drug development.

**Results:**

The dynamic bioreactor is suitable to differentiate hiPSCs in spheroids, which express relevant lung markers in each developmental stage on gene and protein level. The 3D method is able to significantly increase the expression of some markers in comparison to conventional 2D differentiation. 3D-differentiated alveolar epithelial-like cells express functional SARS-CoV-2 receptors and can display the viral infection.

**Conclusion:**

The presented dynamic 3D-differentiation is a promising, new approach to generate alveolar epithelial-like cells from hiPSCs as cell source for in vitro lung models.

**Supplementary Information:**

The online version contains supplementary material available at 10.1007/s44164-023-00052-1.

## Introduction

Lung diseases rank the second place of the global leading causes of mortality. In order to tackle pulmonary diseases and to find new therapeutic options, it is necessary to focus on the development of physiological relevant in vitro lung models. Human induced pluripotent stem cell (hiPSC)-derived alveolar epithelial-like cells (AECs) are promising tools for e.g. pharmaceutical in vitro screening [1]. With regard to the required quality and quantities for this application, the development of reproducible and robust differentiation processes is demanded. Unfortunately, protocols which are suitable for the high-throughput generation of lung cell types from hiPSCs are lacking. To date, hiPSCs have been differentiated into several lung cell types by directed differentiation steps in vitro, simulating the developmental stages of the lung during embryogenesis in vivo [2–7]. Published protocols describing the generation of AECs from pluripotent stem cells mostly contain three-dimensional (3D) differentiation steps, such as embryoid body or organoid formation [3–5]. This creates the impression that the success of differentiating hiPSCs into AECs may depend on how complex developmental pathways can be transferred to in vitro conditions and how the cellular environment, including the interaction with extracellular matrices or the formation of tissue-specific milieus, can be simulated in vitro. These properties can be provided more likely by 3D than two-dimensional (2D) culture conditions [8]. However, 3D-differentiation approaches and organoids are usually very complex, difficult to control regarding the cell composition and nutrient supply. Under static conditions, biomechanical forces that stem cells usually encounter in vivo*,* are not recapitulated in organoids [9–11]. Because of the time-consuming culture and complicated handling procedures, hiPSC-derived lung organoids are not yet applicable to high-throughput approaches such as preclinical drug and toxicity testing in pharmaceutical industry. In this study, a promising approach for the 3D-differentiation of hiPSC-derived alveolar epithelial-like cells type 2 (AEC2) under dynamic conditions is presented. The suspension bioreactor as 3D-differentiation method brings the potential to generate a high amount of AEC2 without many intermediate working steps. The suspension tubes of the bioreactor are moving in alternating directions, keeping the cells under constant movement, providing a homogenous nutrient supply and force the cells to experience shear stress [12]. These are limitations, which static 3D-models and organoids usually entail and probably lead to reproducibility issues. Since it is widely known, that 3D-conditions can simulate in vivo conditions better, than 2D-conditions [8], the new approach has been compared in this study to conventional 2D-conditions within a gene and protein expression analysis of certain key markers during the embryonal lung development. The requirement for this intention was to establish a reproducible differentiation protocol, which works for both culture conditions. For establishing such an uniform protocol, the already published strategies of directed differentiation has been followed [13, 14], but modifications regarding medium composition and differentiation time were necessary in order to achieve the expression of specific markers in both 3D and 2D. Since it is known, that hiPSC experiments suffer from inter-donor variability, meaning that cell lines from different donors also behave different in vitro [15], the comparative analysis was performed with three hiPSC lines from different donors throughout all five stages of lung development. Differences in the marker expression on gene and protein level in the single lung differentiation stages between 2D and dynamic 3D conditions have been determined and the suitability of the suspension bioreactor as 3D differentiation method has been evaluated. As a current example for the application of the derived cells, they were infected with SARS-CoV-2 pseudotypes to proof suitability for their use in COVID-19 drug development.

## Material and methods

### hiPSC maintenance and differentiation

Three hiPSC lines from healthy donors, fully consented for research use and quality controlled, UKKi011-A (SAMEA2774610), UKBi005-A (SAMEA4584351) and BIONi010-C (SAMEA3158050), were obtained from the European Bank for induced pluripotent stem cells (EBiSC) [16] and are also registered on hpscreg.eu. BIONi010-C parental cells were genetically modified using CRISPR/Cas-9 technique by Synthego Inc. F508del mutation of the CFTR protein causing CF was inserted into parental cells and Knock-in clone O16 was isolated, expanded and used in this study as CF disease cell line BIONi010-C-O16. An additional cell line from a 16-year-old male CF patient homozygous for the same mutation, DYP0250 (ACS-1004), was purchased from American Type Culture Collection (ATCC). All hiPSCs were maintained in mTESR1 (STEMCELL) in 60 mm tissue culture plates (Greiner BioOne) coated with growth-factor reduced Matrigel (Corning) according to manufacturer´s instructions. Cultures were routinely passaged once a week using 0.5 mM EDTA in PBS without Ca^2+^ and Mg^2+^ (Fisher Scientific).

### Embryoid body formation in rotating bioreactors

hiPSC at 80–90% confluence were harvested using Accutase (FisherScientific). Digestion was quenched by mTESR1 with 10 µM ROCK inhibitor Y-27632 (ROCK, Tocris) and centrifuged 3 min at 300 g. Cells were resuspended in mTESR1 with 10 µM ROCK. Single cell counting was performed with a NucleoCounter NC-200 (ChemoMetec). 7.5 × 10^5^ viable cells/ mL were transferred in mTESR1 with 10 µM ROCK in Cero Tubes up to 30 mL and placed in the Cero 3D bioreactor (Omni Life Sciences). Usually, 15 × 10^6^ cells were seeded in 20 mL mTESR1 with 10 µM ROCK. Cells were cultivated at 37 °C and 5% CO_2_ under continuous rotation of 60 rpm in alternating directions. After 24 h, Embryoid bodies (EBs) were formed as initial point for the dynamic 3D differentiation.

### Directed differentiation of hiPSC to AECs

Undifferentiated hiPSCs were differentiated via directed differentiation steps in five stages, either under 3D-conditions, starting with 24 h-old EBs or under 2D conditions, starting with hiPSC cultures in petri dishes, as described in the following paragraphs.

### Definitive endoderm (DE)

DE medium (Table 1), supplemented with 1% v/v Penicillin/Streptomycin, was freshly prepared every day. Cells were cultivated for 3 days in DE medium with daily medium exchange. 2D-differentiation was started by feeding 90% confluent hiPSC cultures in 60 mm tissue culture plates with 3 mL DE medium. Usually, two 60 mm culture plates were differentiated per cell line to proceed with one 6 well plate in the next stage (AFE). 3D-differentiation was started by feeding 24 h-old EBs with DE medium. The initially seeded 15 × 10^6^ cells were fed with 15 mL DE medium.

**Table 1:** Differentiation media composition

Stage medium	Base Medium		
DE medium	**RPMI1640** (Fisher Scientific 11530586)	**Activin A** (STEMCELL 78001.1)	**100 ng/ml**
		**CHIR99021** (Sigma Aldrich SML1046-5MG)	**3 µM**
AFE medium	**cSFDM**	**Dorsomorphin** (Sigma Aldrich P5499-5MG)	**2 µM**
		**SB431542** (R&D Systems 1614/10)	**10 µM**
LP medium	**cSFDM**	**CHIR99021** (Sigma Aldrich SML1046-5MG)	**3 µM**
		**BMP4** (Peprotech 120-05ET)	**10 ng/ml**
		**Retinoic acid (RA)** (Sigma Aldrich R2625-50MG)	**0.5 µM**
AEC2 medium	**cSFDM**	**CHIR99021** (Sigma Aldrich SML1046-5MG)	**3 µM**
		**KGF** (Peprotech 100–19)	**10 ng/ml**
		**Dexamethasone** (Alfa Aesar A17590.03)	**50 nM**
		**8-Bromo-cAMP** (Sigma Aldrich B7880-25MG)	**0.1 mM**
		**3-Isobutyl-1-methylxanthine (IBMX)** (Sigma Aldrich I5879-250MG)	**0.1 mM**

### Anterior foregut endoderm (AFE)

Composition of AFE medium was adopted from Jacob et al. [13], consisting of a complete serum free differentiation medium as base medium (cSFDM) (Table 1). In order to establish a uniform protocol working for 2D- and 3D-conditions equally, differentiation time was set to 4 days with medium exchange every other day. 2D-differentiated DE cells were harvested on day 3 by incubation with 0.05% Trypsin/EDTA (FisherScientific) and seeded on freshly Matrigel-coated 6 well plates (Greiner Bio One) at a density of 1.0 × 10^6^ cells per well in AFE medium with 10 µM ROCK (2 mL per well). Medium was exchanged to AFE medium without ROCK on the next day. 3D-differentiated DE spheroids on day 3 were allowed to sink to the bottom. Spent DE medium was aspirated and replaced by AFE medium. The usually seeded cell amount (15 × 10^6^ cells) were fed with 15 mL AFE medium.

### Lung progenitor cells (LP)

Composition of LP medium was adopted from Jacob et al. [13] with changed retinoic acid (RA) concentration (Table 1) in order to facilitate LP differentiation also under 3D-conditions. The cells were fed with LP medium for 9 days with medium exchange every other day. 2D-differentiated AFE cells were harvested on day 7 and reseeded on freshly Matrigel-coated 6 well-plates (GreinerBioOne) at a density of 2 × 10^5^ cells per well in LP medium with 10 µM ROCK (2 mL per well). The medium was exchanged to LP medium without ROCK on the next day. 3D-differentiated AFE spheroids on day 7 were allowed to sink to the bottom. Spent AFE medium was aspirated and replaced by LP medium. The usually seeded cell amount (15 × 10^6^ cells) were fed with 15 mL LP medium.

### Alveolar epithelial-like cells type 2 (AEC2)

The composition of AEC2 medium (Table 1) was adopted from Jacob et al. [13]. 2D-differentiated LP cells were harvested on day 16 and reseeded on freshly Matrigel-coated 6 well-plates at a density of 5 × 10^5^ cells per well in AEC2 medium with 10 µM ROCK (2 mL per well). The medium was exchanged to AEC2 medium without ROCK on the next day. About 12 × 10^6^ AEC2 could be harvested from one 6 well-plate on day 30. 3D-differentiated LP spheroids on day 16 were allowed to sink to the bottom. Spent LP medium was aspirated and replaced by AEC2 medium. Medium was exchanged every 48–72 h for up to 14 days. The usually seeded cell amount (15 × 10^6^ cells) were fed with 20 mL AEC2 medium and resulted in about 100 million cells per tube on day 30.

### Protein expression analysis- flow cytometry

2D-differentiated cells were dissociated using 0.05% Trypsin/EDTA for 5 min at 37 °C. 3D spheroids were dissociated using the Embryoid Body Dissociation Kit (Miltenyi Biotec) according to the manufacturer´s instructions. 1 × 10^6^ cells per sample were fixed and prepared using the Fixation/Permeabilization Kit (BD Biosciences). Samples were stained with 5 µl antibody/million cells for 30 min at 4 °C in staining buffer consisting of 0.1% sodium azide (Sigma Aldrich) and 0.5 µM EDTA (Fisher Scientific) in PBS without Ca^2+^ and Mg^2+^ (Fisher Scientific) supplemented with 2% fetal bovine serum (v/v) (Sigma Aldrich). A list of used antibodies is given in Table S1. Samples were washed once with staining buffer and resuspended in 500 µl fresh staining buffer. Flow cytometry was performed with FACS Calibur (BD Biosciences), using unstained cells as control, drawing the gate by the forward (FSC-A) and side scatter profiling (SSC-A) to define the live cell population and to exclude cell debris. Isotype controls were used for every marker to exclude unspecific antibody bindings by gating the cells of interest. Data analysis was performed using FlowJo V10 software (TreeStar).

### Gene expression analysis- qPCR

2D-differentiated cells were dissociated using 0.05% Trypsin/EDTA for 5 min at 37 °C. 5 × 10^5^ cells per sample were prepared for gene expression analysis using the RNeasy Micro Kit (Qiagen) according to manufacturer´s instructions. 3D spheroids were dissociated using the Embryoid Body Dissociation Kit (Miltenyi Biotec) according to the manufacturer´s instructions.

The RNA concentration was measured using a Nanodrop 2000 (Thermo Fisher Scientific). Reverse transcription was performed using the High Capacity cDNA Reverse Transcription Kit (Applied Biosystems) following the manufacturer´s instructions. Gene expression was measured via the 5´ nuclease assay (TaqMan) quantitative PCR (qPCR) using a Quant Studio 7 Flex system (Applied Biosystems) and TaqMan assays (Fisher Scientific). A list of used TaqMan assays is given in Table S2. Relative quantification (fold change to undifferentiated hiPSCs) was calculated with the 2^−∆∆CT^ method using HPRT1 as endogenous reference for normalization [17]. Human adult lung RNA (Fisher Scientific) was used as reference material to compare the gene expression of hiPSC-derived AEC2 with adult primary lung tissue (both normalized to undifferentiated hiPSCs). Mean values have been calculated by the average of ∆∆CT values of three biological replicates. Based on this, 95% confidence intervals (CI) were calculated with upper and lower limits for the statistical interpretation of the results.

### AEC2 infection with SARS-CoV-2 pseudotypes

SARS-CoV-2 pseudotypes (pCG1-SARS-CoV-2-S/VSVdeltaGFLuc) were generated according to previously published protocol [18]. In brief, 293 T/17 cells transfected with the SARS-CoV-2 viral S-Protein encoding plasmid pCG1-SARS-2-S (kindly provided by Stefan Pöhlmann, Deutsches Primatenzentrum GmbH, Leibniz-Institut für Primatenforschung, Göttingen/Germany) were infected with replication deficient VSV vector (VSV*DG-fLuc, kindly provided by Gert Zimmer, Institute of Virology and Immunology, Mittelhäusern/Switzerland) containing an expression cassette for eGFP and firefly luciferase instead of the VSV-G open reading frame. In order to neutralize residual VSV vector, 293 T/17 cells were washed with PBS and fresh medium supplemented with VSV-G antibody 8G5F11 (Biozol) was added. Infectious titers were calculated by counting GFP-positive cells after the infection of HEK293T-ACE2-TMPRSS2 cells (Genecopoeia) with serial diluted virus supernatant after 48 h. Titers were expressed as fluorescence-forming units per milliliter (ffu/ mL). For infection experiments with AEC2, cells were harvested on day 30. 2D-differentiated cells were dissociated using 0.05% Trypsin/EDTA for 5 min at 37 °C. 3D spheroids were dissociated using the Embryoid Body Dissociation Kit (Miltenyi Biotec) according to the manufacturer´s instructions. Dissociated cells were seeded in Matrigel-coated 96 well-plates (Greiner Bio One) 24 h prior to inoculation with pseudotypes at a density of 1.5 × 10^4^ cells in AEC2 medium with 10 µM ROCK per well. On the day of infection, 50 µL of culture supernatant were discarded and replaced by 2.5 µg/ mL Polybrene (LentiTrans™ Transduction Reagent, Cellecta) in AEC2 medium. 100 µL viral supernatant, equivalent to 6.0 × 10^6^ ffu/mL, were added to the cells and incubated for 72 h at 37 °C. GFP-expression indicating pseudotype-transfected cells were counted using the Leica DMI4000B microscope with Leica application suite software.

### Statistics

For protein expression and pseudotype infection data, values of three biological replicates were averaged and presented as mean ± standard deviation. Unpaired two-tailed Student´s t-test was performed with GraphPad 2022 to test for statistical significance. P-values of 0.05 or lower were considered as statistical significant (***p < 0.001, **p < 0.01,*p < 0.05). Mean values of the gene expression have been calculated by the average of ∆∆CT values of three biological replicates. Based on this, 95% confidence intervals (CI) were calculated with upper and lower limits for the statistical interpretation of the results.

## Results

**Fig. 1:**
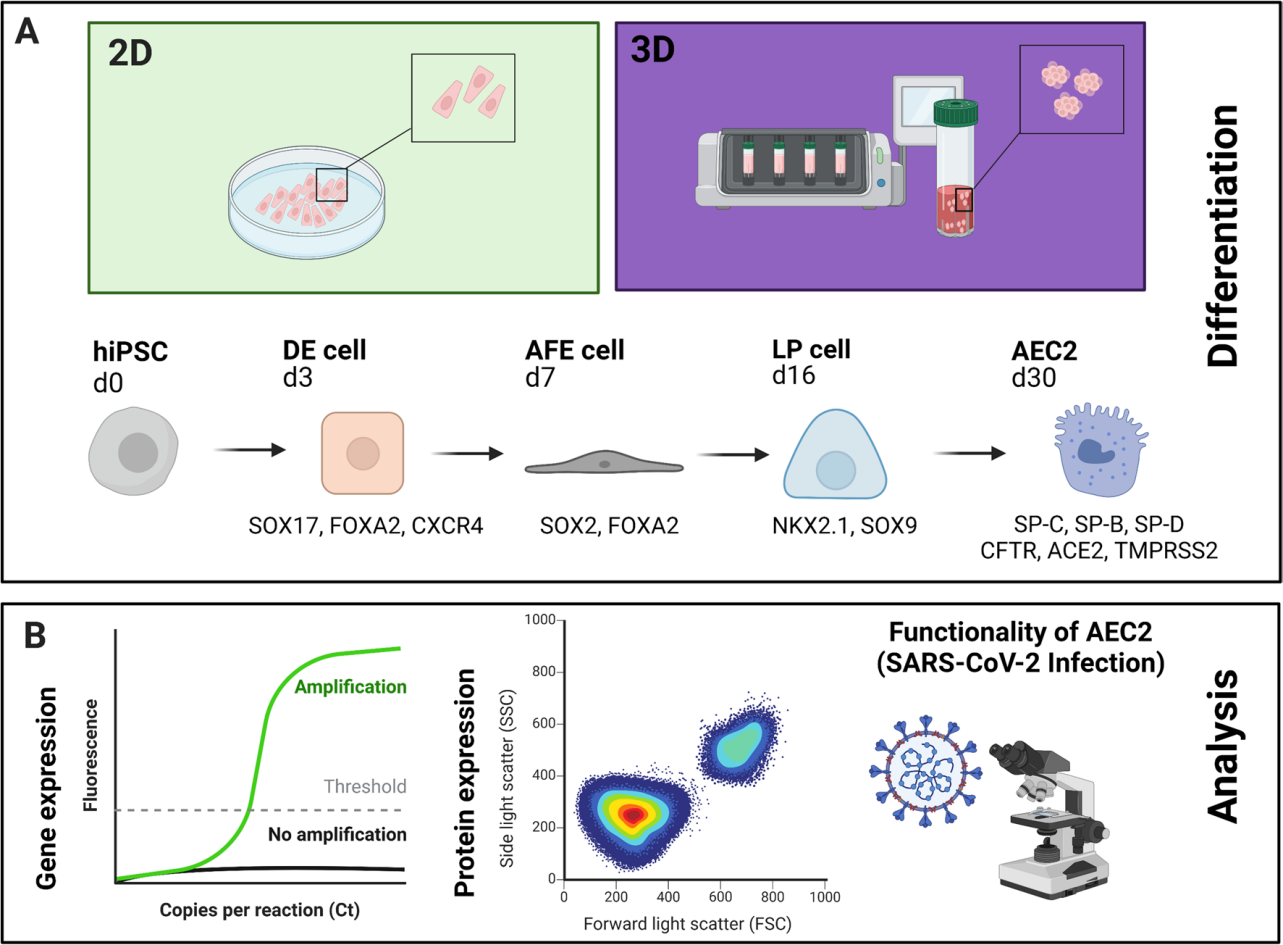
Generation and analysis of human induced pluripotent stem cell (hiPSC)-derived alveolar epithelial-like cells type 2 (AEC2). **A**: AEC2 were generated under 2D-conditions (static) and 3D-conditions (dynamic bioreactor) via directed differentiation within 30 days: Definitive Endoderm (DE), Anterior Foregut Endoderm (AFE), Lung Progenitor cells (LP) and Alveolar-epithelial-like cells type 2 (AEC2). **B**: Analysed endpoints of each differentiation stage (specific pre-selected markers): gene expression analysis by quantitative Real-time PCR (qPCR), protein expression by flow cytometry analysis and verification of cell functionality by infection of AEC2 with fluorescent SARS-CoV-2 pseudoviruses

A new bioreactor-based approach for the hiPSC-differentiation of alveolar epithelial-like cells type 2 (AEC2) under dynamic 3D-conditions has been evaluated in this study in comparison to static conventional 2D-conditions. Three healthy induced pluripotent stem cell lines (UKKi011-A, UKBi005-A and BIONi010-C)[19] have been investigated in five stages of the embryonal lung development (Definitive Endoderm, Anterior Foregut Endoderm, Lung Progenitor Cells, Alveolar epithelial-like cells type 2) (Fig. 1A). Specific markers have been pre-selected for each differentiation stage and determined on gene and protein expression level. Finally, the derived AEC2 were tested for their functionality by SARS-COV-2 pseudotype infection (Fig. 1B).

### 3D-differentiation significantly increases FOXA2 expression in Definitive Endoderm cells

**Fig. 2:**
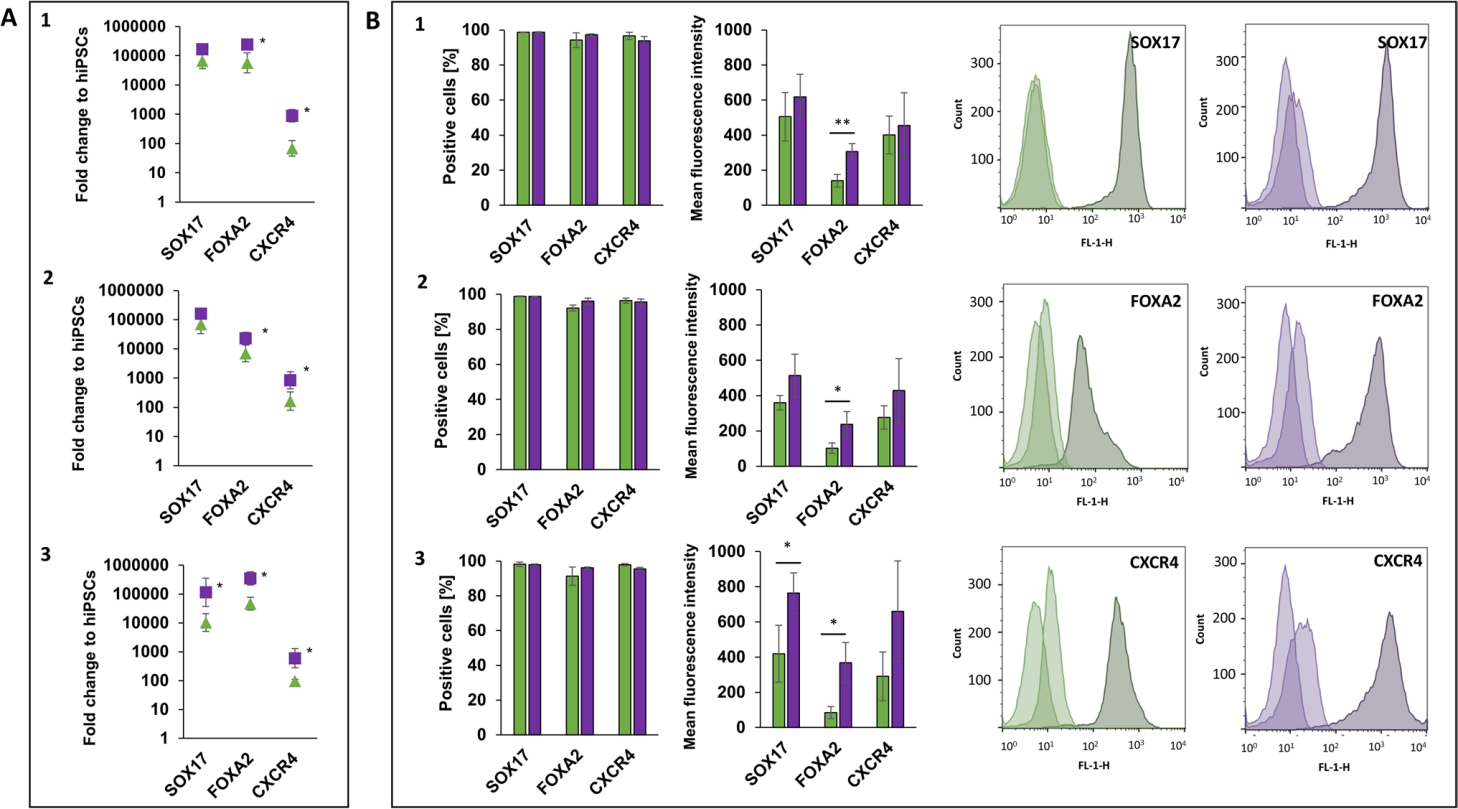
Analysis of Definitive Endoderm (DE) cells on day 3 differentiated from three hiPSC lines from healthy donors under 2D- (green) and 3D- (purple) conditions. **A**: Diagrams of gene expression show the fold change over undifferentiated hiPSCs of the gene of interest analysed by qPCR (2^−∆∆CT^ method). HPRT1 was used as endogenous reference gene. **B**: Protein expression diagrams show the percentage of positive cells and the mean fluorescence intensity for the indicated markers analysed by flow cytometry (representative histograms of cell line 3 BIONi010-C). Data of three independent experiments (gene expression: mean ± CI 95%; *p < 0.05; protein expression: mean ± SD, *p < 0.05, **p < 0.01, unpaired two-tailed Student´s t-test). 1: UKKi011-A, 2: UKBi005-A, 3: BIONi010-C

During the embryonal development in vivo, lung epithelial cells arise from the Definitive Endoderm (DE) germ layer [20]. Within this study, three hiPSC lines from healthy donors have been differentiated under both 2D- and dynamic 3D-conditions into a nearly homogenous population of DE cells within 3 days. They express the endodermal transcription factors SOX17, FOXA2 and CXCR4 on gene and protein level (Fig. 2). On gene level, DE markers are stronger expressed by 3D-differentiated cells than by 2D-differentiated cells (Fig. 2A). Significant differences occur for FOXA2 and CXCR4 in all cell lines and additionally for SOX17 in BIONi010-C (Fig. 2A). Both differentiation approaches give rise to cell populations, which consist of more than 90% positive cells for the DE markers (Fig. 2B). The mean fluorescence intensity (MFI), which correlates to the amount of antibody-presenting antigens in the analysed cells, makes it possible to distinguish the strength of protein expression between the culture conditions. Significant more antigens for FOXA2 were present in 3D- than 2D-differentiated cells (Fig. 2B). Although the gene expression of CXCR4 was significantly higher under 3D conditions in all cell lines, the intensity of CXCR4 protein expression did not differ between the conditions (Fig. 2B). Based on the results, the bioreactor-based approach is suitable to generate a high amount of DE cells expressing all relevant markers on gene and protein level in 3D with the applied protocol. The FOXA2 expression could be significantly increased under this condition.

### FOXA2 expression higher in AFE stage under 3D-differentiation conditions

The patterning of the anterior foregut endoderm (AFE), from which the embryonal lung arises, follows the DE. AFE cells are characterized by the expression of SOX2, a pluripotency marker of hiPSCs that re-emerges in this stage as differentiation marker, while the expression of FOXA2 is maintained [14]. Both markers have been analysed in this stage on gene and protein expression level to determine the differentiation efficacy of the applied methods.

**Fig. 3:**
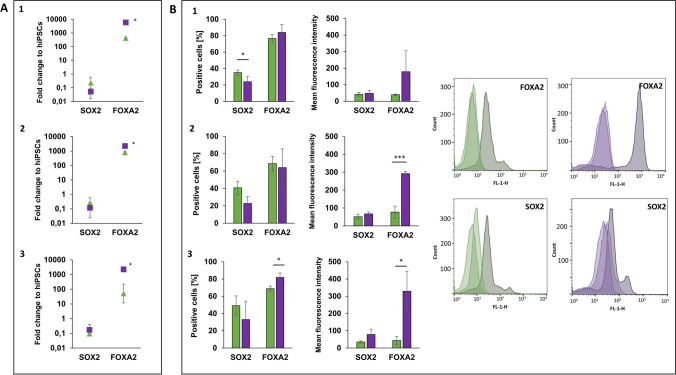
Analysis of Anterior Foregut Endoderm (AFE) cells on day 7 differentiated from three hiPSC lines from healthy donors under 2D (green) and 3D (purple) conditions. **A**: Diagrams of gene expression show the fold change over undifferentiated hiPSCs of the gene of interest analysed by qPCR (2^−∆∆CT^ method). HPRT1 was used as endogenous reference gene. **B:** Protein expression diagrams show the percentage of positive cells and the mean fluorescence intensity for the indicated markers analysed by flow cytometry (representative histograms of cell line 3 BIONi010-C). Data of three independent experiments (gene expression: mean ± CI 95%; *p < 0.05; protein expression: mean ± SD, *p < 0.05, ***p < 0.001 unpaired two-tailed Student´s t-test). 1: UKKi011-A, 2: UKBi005-A, 3: BIONi010-C

SOX2 could be detected in all cell lines under both differentiation conditions, but is weaker expressed by AFE cells on gene level than by undifferentiated hiPSCs, indicated by the fold change smaller than 1 (Fig. 3A). This is not surprisingly, since pluripotency markers are overexpressed in hiPSCs. Less than half of the analysed cells express SOX2 protein under both 2D- and 3D-conditions (Fig. 3B), with a tendency to be higher in 2D (significant in one of the three cell lines) (Fig. 3B-1). However, MFI revealed that those cells must express SOX2 weaker than their 3D-differentiated counterparts, since the MFI did not differ between the conditions (Fig. 3B-1). The FOXA2 gene expression in AFE cells is again significantly higher under 3D-conditions in all investigated cell lines (Fig. 3A). Significant more AFE cells differentiated from BIONi010-C express FOXA2 on protein level more intensively, which shows the significant higher MFI (Fig. 3B-3). The MFI of FOXA2 in UKBi005-A is also significantly higher in 3D, even though the amount of positive cells in the analysed cell population did not differ to 2D (Fig. 3B-2). In conclusion, the 3D-approach is suitable to differentiate AFE cells with a significant higher FOXA2 expression than under 2D-conditions.

### 2D- and 3D-differentiated lung progenitor cells are both NKX2.1^+^ and SOX9^+^

**Fig. 4:**
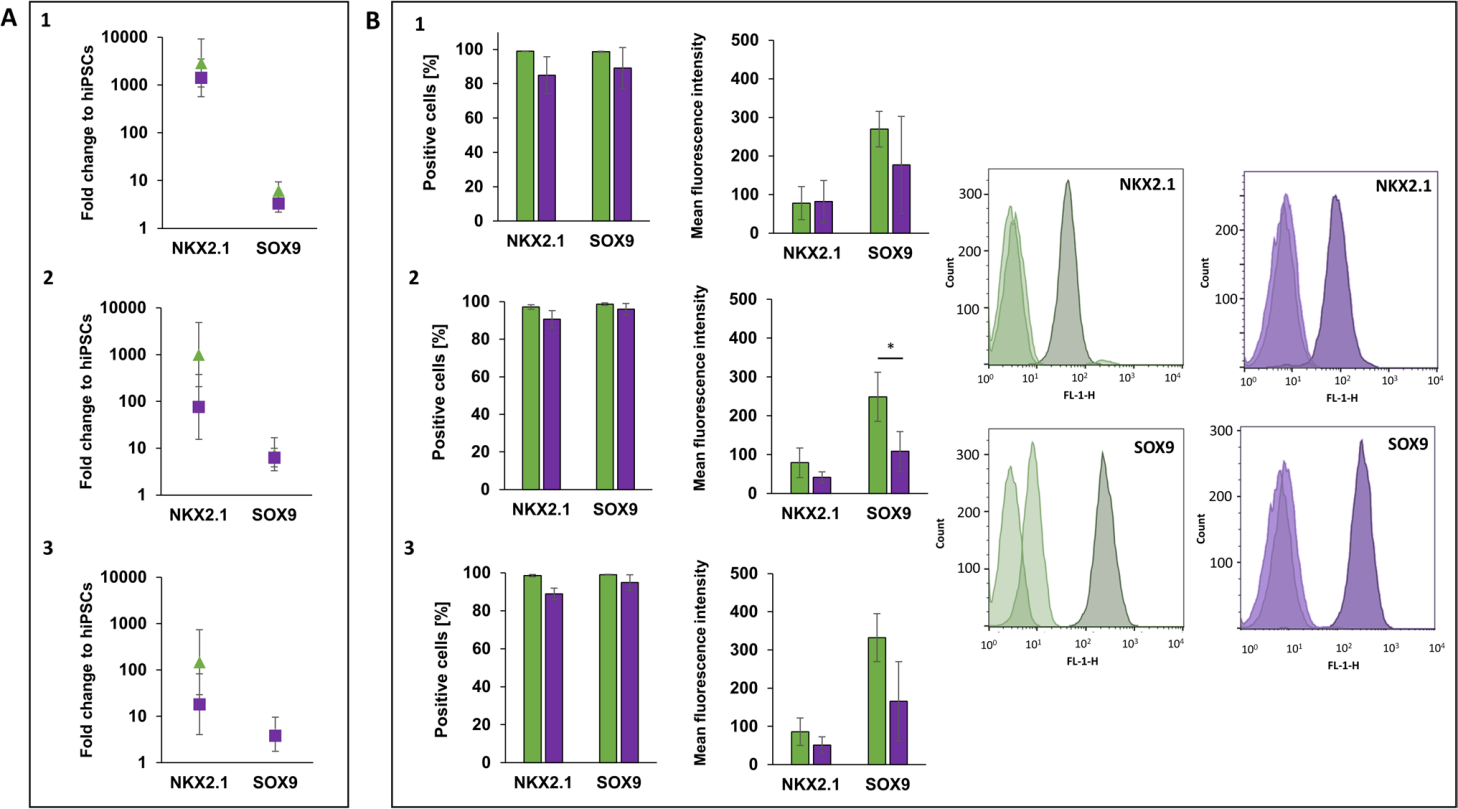
Analysis of Lung Progenitor (LP) cells on day 16 differentiated from three hiPSC lines from healthy donors under 2D- (green) and 3D- (purple) conditions. **A**: Diagrams of gene expression show the fold change over undifferentiated hiPSCs of the gene of interest analysed by qPCR (2^−∆∆CT^ method). HPRT1 was used as endogenous reference gene. **B:** Protein expression diagrams show the percentage of positive cells and the mean fluorescence intensity for the indicated markers analysed by flow cytometry (representative histograms of cell line 3 BIONi010-C). Data of three independent experiments (gene expression: mean ± CI 95%; *p < 0.05; protein expression: mean ± SD, *p < 0.05, unpaired two-tailed Student´s t-test). 1: UKKi011-A, 2: UKBi005-A, 3: BIONi010-C

After AFE-patterning, the lung primordium is formed along the ventral axis and expresses the lung fate marker NKX2.1. NKX2.1^+^ lung progenitors give rise to SOX9^+^ cells, which finally differentiate into alveolar cell types [21]. Thus, NKX2.1 is the first relevant key marker, which is connected to lung fate and SOX9 is the first marker, which relates to distal specification. Both markers are the quality criteria of a successful ongoing lung progenitor (LP) differentiation in 2D and 3D and were analysed on gene and protein level in three different cell lines in this study. On gene expression level, no significant differences occur for both markers between the culture conditions (Fig. 4A). The percentage of NKX2.1^+^ and SOX9^+^ cells was not significantly different between 2D- and 3D-differentiation conditions (Fig. 4B). Both methods were suitable to generate a cell population consisting of more than 98.4% NKX2.1^+^ and 99.2% SOX9^+^ cells in 2D and 88.9% NKX2.1^+^ and 92.2% SOX9^+^ cells in 3D (Fig. 4B). Significant differences in the MFI only occurred for SOX9 in UKBi005-A, in which the intensity of SOX9 was higher in 2D-differentiated LP cells (Fig. 4B-2). Thus, within this stage of differentiation, it has been observed that 3D-conditions did not increase the gene expression in comparison to 2D, as it has been observed in the previous stages. Especially NKX2.1 gene actually seems to be higher expressed under 2D-conditions. Nevertheless, on protein level, both culture conditions lead to almost homogenous cell populations, which are both positive for the lung fate markers NKX2.1 and SOX9.

### AEC2-marker expressing cells are present after 23 days under 2D- and 3D-differentiation conditions

**Fig. 5:**
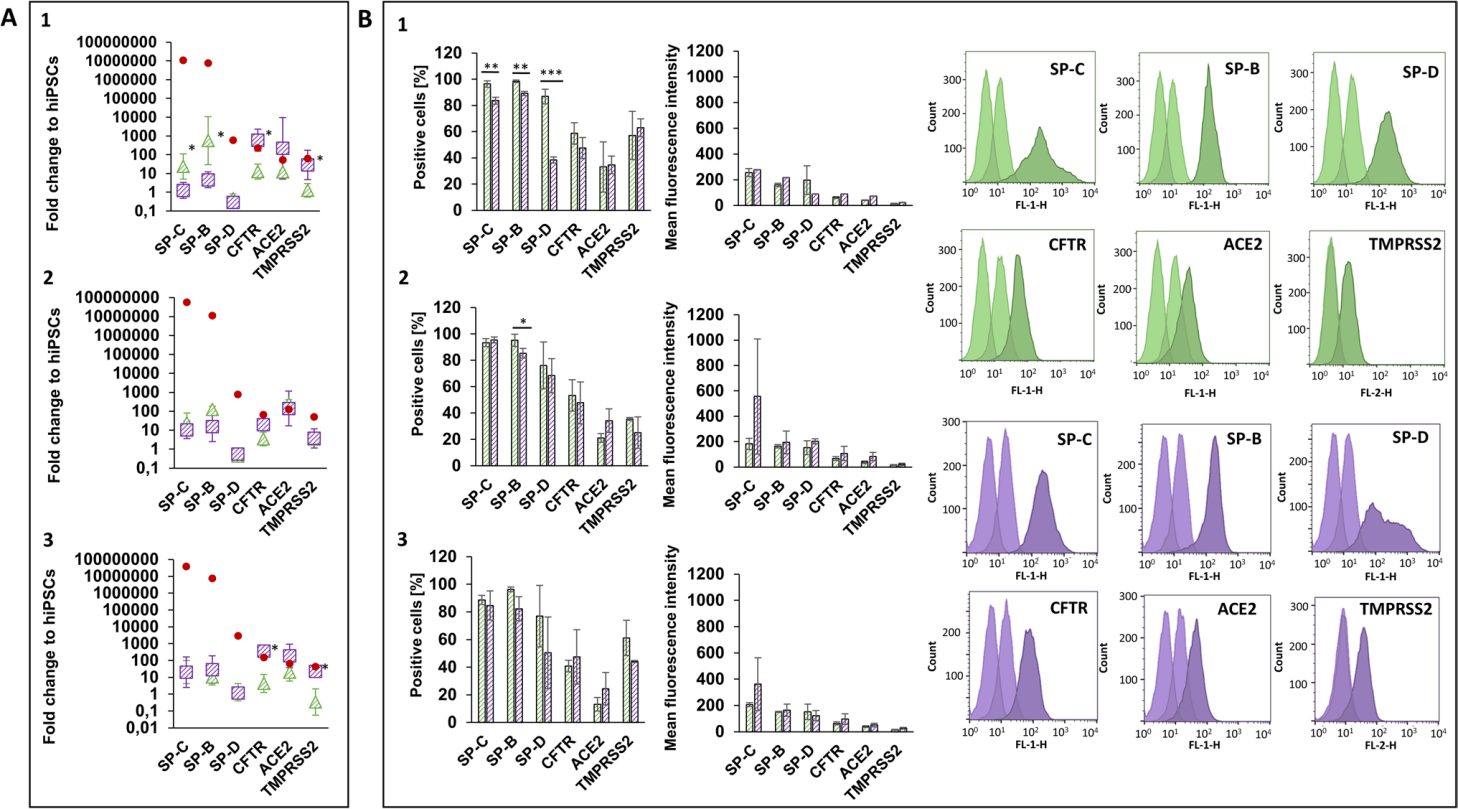
Analysis of Alveolar epithelial-like cells type 2 (AEC2) on day 23 differentiated from three hiPSC lines from healthy donors under 2D- (green) and 3D- (purple) conditions. **A**: Diagrams of gene expression show the fold change over undifferentiated hiPSCs of the gene of interest analysed by qPCR (2^−∆∆CT^ method). HPRT1 was used as endogenous reference gene. Red dots show gene expression of respective markers in adult human lung. **B**: Protein expression diagrams show the percentage of positive cells and the mean fluorescence intensity for the indicated markers analysed by flow cytometry (representative histograms of cell line 3 BIONi010-C). Data of three independent experiments (gene expression: mean ± CI 95%; *p < 0.05; protein expression: mean ± SD, *p < 0.05, **p < 0.01, ***p < 0.001, unpaired two-tailed Student´s t-test). 1: UKKi011-A, 2: UKBi005-A, 3: BIONi010-C

**Fig. 6:**
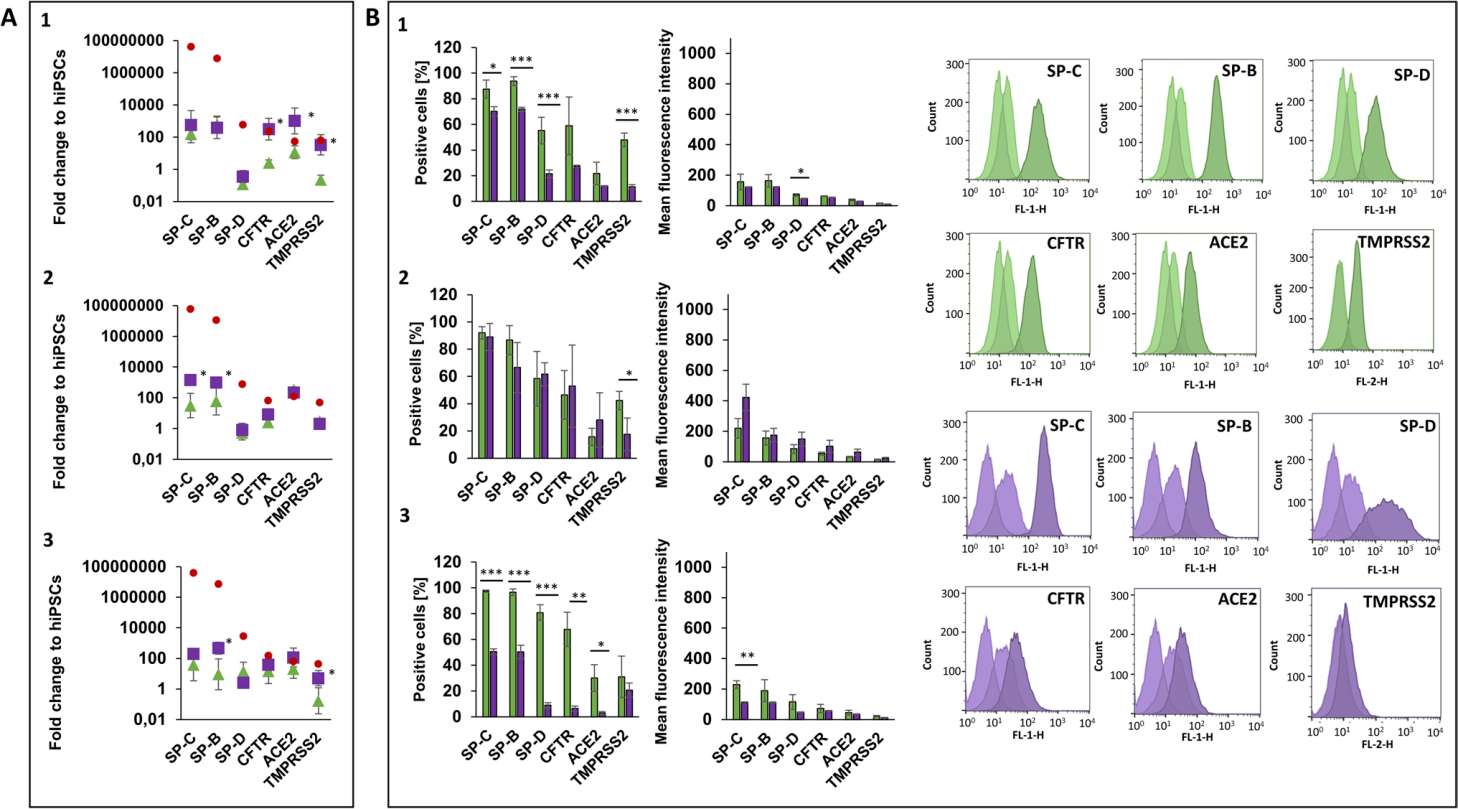
Analysis of Alveolar epithelial-like cells type 2 (AEC2) on day 30 differentiated from three hiPSC lines from healthy donors under 2D (green) and 3D (purple) conditions. **A**: Diagrams of gene expression show the fold change over undifferentiated hiPSCs of the gene of interest analysed by qPCR (2^−∆∆CT^ method). HPRT1 was used as endogenous reference gene. Red dots show gene expression of respective markers in adult human lung. **B**: Protein expression diagrams show the percentage of positive cells and the mean fluorescence intensity for the indicated markers analysed by flow cytometry (representative histograms of cell line 3 BIONi010-C). Data of three independent experiments (gene expression: mean ± CI 95%; *p < 0.05; protein expression: mean ± SD, *p < 0.05, **p < 0.01, ***p < 0.001, unpaired two-tailed Student´s t-test). 1: UKKi011-A, 2: UKBi005-A, 3: BIONi010-C

LP cells were further differentiated into AEC2 until 30 days of total differentiation time. Gene and protein expression analyses of AEC2 have been performed on day 23 as intermediate time point (Fig. 5) and on day 30 (Fig. 6). LP cells developed into cell populations, which express typical alveolar markers such as surfactant protein (SP)-C, which is uniquely expressed by alveolar epithelial cells type 2 in the human body [22], SP-B and SP-D, as well as the membrane receptors targeted by SARS-CoV-2, angiotensin converting enzyme 2 (ACE2) and transmembrane serine protease 2 (TMPRSS2), which have been identified to be expressed by alveolar epithelial cells type 2 [23]. Additionally, the epithelial cell marker Cystic Fibrosis (CF) Transmembrane Conductance Regulator (CFTR), which plays also a role in the pathogenesis of CF [24], has been determined on the surface of 2D- and 3D-differentiated AEC2 in this study (Figs. 5 and 6). The presence of all pre-selected markers was proven on day 23 and 30 on gene and protein level (Figs. 5 and 6). Differences in the strength of expression were found between the two time points. Gene expression of SP-C and SP-B increased from day 23 to 30 to up to 400-fold, whereas SP-D and ACE2 stayed at the same level or just increased slightly and CFTR and TMPRSS2 decreased from day 23 to 30 (Figs. 5A and 6A). Many of the analysed markers showed a significant higher gene expression under 3D-differentiation conditions, such as CFTR and TMPRSS2 in UKKi011-A (Fig. 5A-1) and BIONi010-C (Fig. 5A-3). On day 30, TMPRSS2 was higher expressed in 3D in UKKi011-A (Fig. 6A-1) and BIONi010-C (Fig. 6A-3), SP-B in UKBi005-A and BIONi010-C, SP-C in UKBi005-A and CFTR and ACE2 in UKKi011-A (Fig. 6A). A stronger gene expression in 2D-differentiated cells was observed on day 23 for SP-C and SP-B in UKKi011-A (Fig. 5A-1). There is still a huge disparity between the SP expression in human adult lung RNA, which was analysed as reference and AEC2 derived from hiPSCs of about 200.000-fold. Regarding CFTR, ACE2 and TMPRSS2, the gene expression of the analysed AEC2 is on a comparable level to human lung (Figs. 5A and 6A).

The percentage of AEC2 marker expressing cells on day 23 varied between 2D- and 3D-differentiated cells for SP-C in one, for SP-B in two and for SP-D in one out of three cell lines. The intensity of protein expression (MFI) of all analysed markers in all cell lines did not distinguish on day 23 (Fig. 5B). On day 30, more significant differences occurred on protein level. All analysed surfactant proteins (SP-C, SP-B and SP-D) were expressed by significant more 2D-differentiated cells in UKKi011-A (Fig. 6B-1) and BIONi010-C cell line (Fig. 6B-3). Immuncytochemistry (ICC) staining of SP-C in 2D- and 3D-differentiated AEC2 is shown in Fig. S1. ICC staining of the 2D- and 3D-differentiated healthy cell lines into AEC2 enabled to identify varying cell morphologies between the cell lines in 2D, indicated by the different shapes of nuclei, diverse structures of actin filaments and dissimilar distribution of SP-C (Fig. S1). Such differences could not be observed in 3D-differentiated cell spheres. In comparison to 2D, nuclei had comparable shapes, actin filaments were more compressed and SP-C seemed to be distributed more homogenously in 3D (Fig. S1). Additionally, CFTR and ACE2 were expressed by significant more BIONi010-C cells differentiated under 2D (Fig. 6B-3). TMPRSS2 was expressed by significant more 2D-differentiated UKKi011-A (Fig. 6B-1) and UKBi005-A cells (Fig. 6B-2). The MFI demonstrate that more antigens for SP-C were detected on 2D-differentiated BIONi010-C (Fig. 6B-3) and for SP-D on more 2D-differentiated UKKi011-A cells (Fig. 6B-1). Comparing both time points, it was clearly identified, that gene expression of the markers was higher on day 30 and protein expression was higher on day 23. Whereas protein expression stayed the same or increased from day 23 to 30 in 2D, it decreased in 3D (Figs. 5B and 6B). The 2D- and 3D-differentiation and comparative analysis has been performed in this study also with Cystic Fibrosis (CF) hiPSC lines carrying the F508del mutation. The results are comparable to those of healthy cell lines except for the SP-C and SP-B gene expression in DYP0250, which was quite low (Figs. S2 and S3). However, the cells expressed all markers on protein level and can be used to establish CF disease models in future. More staining and imaging of the 3D-differentiated cell populations must be performed in future studies, to characterize and identify the maturity of the resulting cell types of this differentiation protocol in detail. As a first step, these results demonstrate the potential and suitability of the dynamic bioreactor, to generate alveolar epithelial-like cells under 3D conditions from hiPSCs.

### 2D- and 3D-differentiated AEC2 are both suitable to model in vitro infection with SARS-CoV-2 pseudotypes

**Fig. 7:**
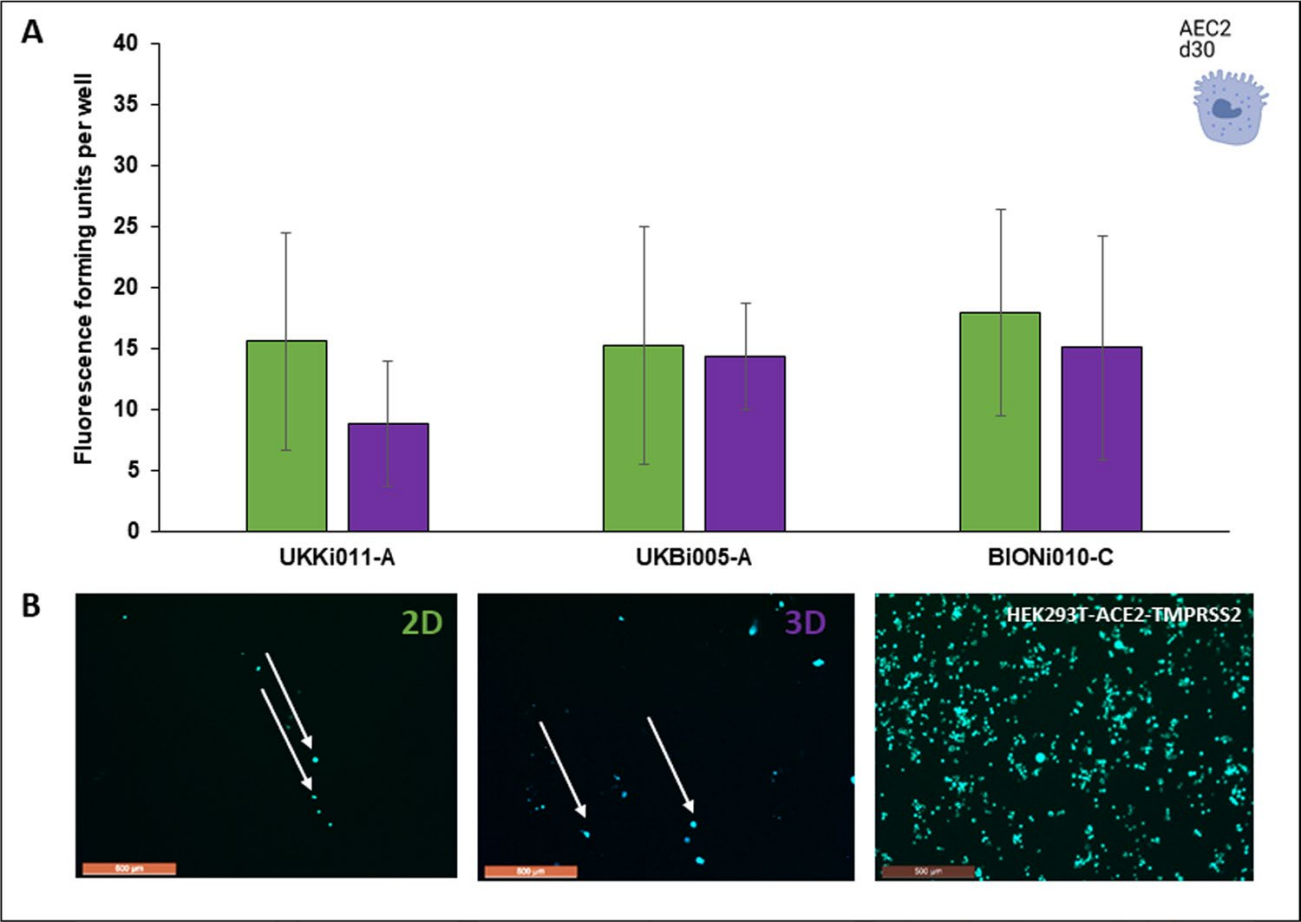
Infection of AEC2 on day 30 with fluorescent SARS-CoV-2 pseudotypes.** A**: Diagram represents the absolute number of fluorescence-forming units (ffu) per well of three different cell lines, differentiated into in either 2D (green) or 3D (purple). Data of three independent experiments (mean ± SD, not significant, unpaired two-tailed Student´s t-test). **B**: Representative images of ffu (indicated by white arrows) in cultures from 2D- and 3D-diffferentiated AEC2 as well as HEK293T-ACE2-TMPRSS2 as positive control for pseudotype infection

Recently, it has been revealed that SARS-CoV-2 infection depends on the host cell factors ACE2 and TMPRSS2 [18]. AEC2 have been identified as the cell type, which expresses both factors in the distal lung. Spike proteins of SARS-CoV-2 bind to ACE2 as entry receptor on the target cell surface and utilize TMPRSS2 for the spike protein priming, which allows fusion of viral and cellular membranes [25]. For the future application of hiPSC-derived AEC2 for e.g. drug development studies for COVID-19 or viral neutralization experiments, the suitability of the derived cell types and the functionality of the expressed target receptors should be demonstrated. AEC2, which have been generated in this study under 2D- and 3D-conditions, express SARS-CoV-2 receptors on gene and protein level regardless of their differentiation condition (Figs. 5 and 6). In a next step, we produced SARS-CoV-2 pseudotypes with a replication-deficient VSV vector that contains expression cassettes for GFP (green fluorescent protein) according to Hoffmann et al. [18], which enables to identify pseudotype-infected cells by fluorescence. It could be proven, that 2D- and 3D-differentiated AEC2 possess functional SARS-CoV-2 host cell factors on their cell surface, since infected cells could be determined in each of the cell populations independently from their differentiation condition and cell line (Fig. 7). Significant differences in the number of fluorescence-forming units (ffu) between 2D- and 3D-differentiated cells could not be observed (Fig. 7A). HEK293T-ACE2-TMPRSS2 cell line, which was conducted as a positive control cell line and is currently used as cell model in viral neutralization studies, was significantly more infected than hiPSC-derived AEC2 (Fig. 7B).

## Discussion

Reproducible and stable protocols for the differentiation of hiPSCs in alveolar epithelial cells (AECs) are highly demanded, since their application seems very promising for toxicological and pharmaceutical in vitro screenings [1]. Unfortunately, protocols which are suitable for the high-throughput generation of AECs from hiPSCs are lacking. The primarily objective of this study was the presentation of a new, promising approach for the hiPSC-differentiation in AEC2 under dynamic 3D-conditions. From our point of view, it is the method of choice when it comes to the implementation of hiPSC-derived (lung) cell types in drug development and toxicity testing. On the one hand, 3D and organoid models are too time-consuming and low-throughput in handling, that there is no chance for a broad application yet [9, 10]. On the other hand, no 2D monolayer-like differentiation protocol has been reported except for one recent publication describing a differentiation strategy for AEC2 in a 96-well format at air–liquid-interface (ALI) [26]. This was the first study, which also pursued for the practical application of this promising cell source in industry. The use of the suspension bioreactor as 3D-differentiation method helps to overcome several limitations of existing 3D-protocols. For evaluating this, the differentiation efficacy has been determined within a gene and protein expression analysis of certain key markers during the embryonal lung development in comparison to conventional 2D-conditions.

Within the first two differentiation stages, DE and AFE, differences between 2D and 3D have been most clearly, especially regarding the expression of the transcription factor FOXA2. Since FOXA2 was significant higher in both gene and protein expression, it can be concluded that FOXA2 in DE and AFE stage is stronger activated under dynamic 3D-conditions. This may be because of shear stress, which embryonic cells encounter in vivo. Furthermore, cells in 3D are ordered in a completely different manner, so that stem cell niches can be better simulated [8]. Such significant differences could not been observed in any of the following stages. Differentiating LP cells, which are positive for the early lung marker NKX2.1 has been described as challenging and often varying from 20–90% NKX2.1^+^ cells, as published previously [13]. In our study, almost homogenous populations with more than 90% NKX2.1^+^ cells could be generated independently from the used cell line and culture condition, which may be explained by the increased concentration of retinoic acid (RA) in the LP medium, which has been identified in this study to enable the successful differentiation of LP cells under dynamic 3D-conditions. Concurrently, LP cells were identified to be also about 90% positive for SOX9, thus serving as an ideal starting population for the distal maturation, since SOX9^+^ cells are distal progenitors [1, 27]. The effective differentiation into AEC2 could be demonstrated by the expression of SP-C, SP-B and SP-D, as well as CFTR, ACE2 and TMPRSS2. This proved, that a long-time cultivation and pre-sorting of LP cells is not necessary to get AEC2-marker positive cells in a 3D model. A strong disparity, however, could be observed in this study between hiPSC-derived AEC2 and adult human lung on gene expression level. This may be explained by the alveolar formation and maturation process, which is ongoing from 36 weeks of gestation up to three years after birth. It is even suggested that alveolar formation takes up to the young adulthood in vivo [28], which is difficult to simulate under in vitro conditions within a period of a few weeks. Therefore it is assumed, that hiPSC-derived lung cell types more resemble fetal human lung than adult human lung [3]. The observed differences in the gene expression regarding surfactant proteins between human adult lung RNA and hiPSC-derived AEC2 confirm this assumption again. In many other studies, marker expression is often compared to fetal human or murine lung [2, 3, 5], instead of adult human lung. Additionally, after birth, alveoli are expanding the first time and are exposed to air. Therefore, a higher expression of surfactant proteins and a more advanced maturation is likely to be achieved at ALI culture [26, 29–31]. The membrane receptors CFTR, ACE2 and TMPRSS2 were conversely expressed on a comparable level to adult human lung, assuming that these markers may be in a quite matured state already in fetal lung. The protein expression data from AEC2 on day 23 and 30 indicate, that the cultivation time has a crucial influence on the protein expression in 2D and 3D. Whereas in 2D the protein expression increased or stayed at the same level, it decreased in 3D. However, the gene expression in 3D was higher on day 30, indicating that protein synthesis is still ongoing and this coincides with the knowledge that 2D cultures differentiate faster than 3D cultures [8]. It has to be noted that gene and protein expression are time-shifted and continuously ongoing processes, when interpreting single time-points. The two selected time points in the alveolar stage in this study helped to identify the influence of timing on the differentiation success, also the selection of cell lines influences the result. Therefore, the needed cultivation time for this 3D-differentiation approach should be determined and set individually for each cell line and the researcher´s needs. Although it is assumed, that AEC2 need more maturation time under 3D-conditions, the bioreactor brings the outstanding advantage to produce a higher cell mass with fewer working steps than under 2D-conditions. In comparison to the 2D-differentiation, which requires the harvesting and reseeding of the cells in every differentiation stage, the bioreactor-based 3D-differentiation allows to produce AEC2 with significant fewer handling procedures. Harvesting and reseeding are no longer necessary, only media exchanges are still part of the working process. Therefore, a large-scale production (about 400 million) AEC2 for pharmaceutical industry is feasible with one bioreactor batch. Furthermore, it can be decided individually, if the cells are getting used as currently requested 3D models (spheroids) or they need to be dissociated in single cell populations. Once the process should be up-scaled and larger culture vessels are needed, it is required to achieve the same dynamic conditions, since shear stress is a crucial factor on the homogenous nutrient supply and hence on the differentiation success and reproducibility.

Since AEC2 have been defined as target cells for SARS-CoV-2, they are requested cell types to date in order to test drugs for the treatment of COVID-19 or to inhibit viral entry [25]. The infection of hiPSC-derived AEC2 with living SARS-CoV-2 at ALI has been published recently [32]. We were also able to demonstrate that 2D- and dynamic 3D-differentiated AEC2 in this study could be infected with SARS-CoV-2 pseudotypes. In a previous study by Zhao et al. [33], primary AEC2 have been found to be 1.4 ± 0.4% positive for AEC2. In our study, 20.7 ± 6.8% of 2D-differentiated AEC2 and 13.2 ± 9.4% of 3D-differentiated AEC2 were ACE2^+^ on day 30. Although obviously more hiPSC-derived AEC2 express ACE2 than in vivo, the number of infected cells remained low in comparison to the transfected cell line HEK293T-ACE2-TMPRSS2 cells, meaning that the physiological situation is resembled more realistic by hiPSC-derived AEC2 than by transfected cell lines. In conclusion, the dynamic 3D-differentiation is a method with a high potential to generate functional AEC2 in high numbers within 30 days for e.g. SARS-CoV-2 studies. It has also been shown in this study, that hiPSC lines with CF mutations are suitable to differentiate into AEC2, meaning that the presented approach is promising to generate lung cell types with disease-specific phenotypes to establish reliable lung disease models in future.

## Conclusion

The dynamic 3D-approach is very promising to reduce reproducibility issues of 3D-differentiation protocols and to generate a high amount of AEC2 within a spheroid, which can either be used directly as 3D-model or as a stable cell source for establishing ALI models in future. Significant differences occurred in the first two stages of alveolar differentiation: The FOXA2 expression in DE and AFE stage was higher under 3D conditions. Even though single differences have been observed also in the later stages, they were not observed in every hiPSC line used and therefore it cannot be traced back solely to the differentiation condition. Another important result of this study is the knowledge, that the 2D differentiation is also suitable to generate AECs, which express relevant markers throughout the lung development. Finally, we demonstrated the suitability of the dynamic 3D-differentiation approach to generate high amounts of AECs suitable for e.g. SARS-CoV-2 studies.

## Supplementary Information

Below is the link to the electronic supplementary material.

Supplementary file1 (DOCX 2074 KB)

## Data Availability

The data that support the findings of this study are available from the corresponding author upon reasonable request.

## References

[1] Moreira A, Müller M, Costa PF, Kohl Y. Advanced in vitro lung models for drug and toxicity screening: the promising role of induced pluripotent stem cells. Adv Biol. 2021;6:2101139. 10.1002/adbi.202101139. 10.1002/adbi.202101139 34962104

[2] Gotoh S, Ito I, Nagasaki T, Yamamoto Y, Konishi S, Korogi Y, Matsumoto H, Muro S, Hirai T, Funato M, Mae SI, Toyoda T, Sato-Otsubo A, Ogawa S, Osafune K, Mishima M. Generation of alveolar epithelial spheroids via isolated progenitor cells from human pluripotent stem cells. Stem Cell Reports. 2014;3(3):394–403. 10.1016/J.STEMCR.2014.07.005. 25241738 10.1016/j.stemcr.2014.07.005 PMC4266003

[3] Dye BR, Hill DR, Ferguson MA, Tsai YH, Nagy MS, Dyal R, Wells JM, Mayhew CN, Nattiv R, Klein OD, White ES, Deutsch GH, Spence JR. In vitro generation of human pluripotent stem cell derived lung organoids. Elife. 2015;4(4):1–25. 10.7554/ELIFE.05098. 10.7554/eLife.05098 PMC4370217 25803487

[4] Yamamoto Y, Korogi Y, Hirai T, Gotoh S. A method of generating alveolar organoids using human pluripotent stem cells. Methods Cell Biol. 2020;159:115–41. 10.1016/BS.MCB.2020.02.004. 32586440 10.1016/bs.mcb.2020.02.004

[5] Jacob A, Morley M, Hawkins F, McCauley KB, Jean JC, Heins H, Na CL, Weaver TE, Vedaie M, Hurley K, Hinds A, Russo SJ, Kook S, Zacharias W, Ochs M, Traber K, Quinton LJ, Crane A, Davis BR, White FV, Wambach J, Whitsett JA, Cole FS, Morrisey EE, Guttentag SH, Beers MF, Kotton DN. Differentiation of human pluripotent stem cells into functional lung alveolar epithelial cells. Cell Stem Cell. 2017;21(4):472-488.e10. 10.1016/J.STEM.2017.08.014. 28965766 10.1016/j.stem.2017.08.014 PMC5755620

[6] Huang SXL, Islam MN, O’Neill J, Hu Z, Yang YG, Chen YW, Mumau M, Green MD, Vunjak-Novakovic G, Bhattacharya J, Snoeck HW. Highly efficient generation of airway and lung epithelial cells from human pluripotent stem cells. Nat Biotechnol. 2014;32(1):84. 10.1038/NBT.2754. 24291815 10.1038/nbt.2754 PMC4101921

[7] Kanagaki S, Ikeo S, Suezawa T, Yamamoto Y, Seki M, Hirai T, Hagiwara M, Suzuki Y, Gotoh S. Directed induction of alveolar type I cells derived from pluripotent stem cells via Wnt signaling inhibition. Stem Cells (Dayton, Ohio). 2021;39(2):156–69. 10.1002/STEM.3302. 33241896 10.1002/stem.3302 PMC7898721

[8] Duval K, Grover H, Han LH, Mou Y, Pegoraro AF, Fredberg J, Chen Z. Modeling physiological events in 2D vs. 3D cell culture. Physiology. 2017;32(4):266. 10.1152/PHYSIOL.00036.2016. 28615311 10.1152/physiol.00036.2016 PMC5545611

[9] Corrò C, Novellasdemunt L, Li VSW. Making cell culture more physiological: a brief history of organoids. Am J Phys Cell Physiol. 2020;319(1):C151. 10.1152/AJPCELL.00120.2020. 10.1152/ajpcell.00120.2020 PMC7468890 32459504

[10] Fatehullah A, Tan SH, Barker N. Organoids as an in vitro model of human development and disease. Nat Cell Biol. 2016;18(3):246–54. 10.1038/ncb3312. 26911908 10.1038/ncb3312

[11] Hofer M, Lutolf MP. Engineering organoids. Nat Rev Mater. 2021;6(5):402. 10.1038/S41578-021-00279-Y. 33623712 10.1038/s41578-021-00279-y PMC7893133

[12] Keong Kwok C, Sébastien I, Hariharan K, Meiser I, Wihan J, Altmaier S, Karnatz I, Feile A, Cabrera-Socorro A, Rasmussen M, Holst B, Neubauer JC, Clausen C, Verfaillie C, Ebneth A, Hansson M, Steeg R, Zimmermann H. Scalable expansion of iPSC and their derivatives across multiple lineages. Reprod Toxicol. 2022;112:23–35. 35595152 10.1016/j.reprotox.2022.05.007

[13] Jacob A, Vedaie M, Roberts DA, Thomas DC, Villacorta-Martin C, Alysandratos KD, Hawkins F, Kotton DN. Derivation of self-renewing lung alveolar epithelial type II cells from human pluripotent stem cells. Nat Protoc. 2019;14(12):3303–32. 10.1038/s41596-019-0220-0. 31732721 10.1038/s41596-019-0220-0 PMC7275645

[14] Green MD, Chen A, Nostro MC, D’Souza SL, Schaniel C, Lemischka IR, Gouon-Evans V, Keller G, Snoeck HW. Generation of anterior foregut endoderm from human embryonic and induced pluripotent stem cells. Nat Biotechnol. 2011;29(3):267. 10.1038/NBT.1788. 21358635 10.1038/nbt.1788 PMC4866999

[15] Volpato V, Webber C. Addressing variability in iPSC-derived models of human disease: guidelines to promote reproducibility. Disease Models & Mechanisms. 2020;13:dmm042317. 10.1242/DMM.042317. 31953356 10.1242/dmm.042317 PMC6994963

[16] Steeg R, Neubauer JC, Müller SC, Ebneth A, Zimmermann H. The EBiSC iPSC bank for disease studies. Stem Cell Res. 2020;49:102034. 10.1016/J.SCR.2020.102034. 33099110 10.1016/j.scr.2020.102034

[17] Livak KJ, Schmittgen TD. Analysis of relative gene expression data using real-time quantitative PCR and the 2(-Delta Delta C(T)) Method. Methods (San Diego, Calif). 2001;25(4):402–8. 10.1006/METH.2001.1262. 11846609 10.1006/meth.2001.1262

[18] Hoffmann M, Kleine-Weber H, Schroeder S, Krüger N, Herrler T, Erichsen S, Schiergens TS, Herrler G, Wu NH, Nitsche A, Müller MA, Drosten C, Pöhlmann S. SARS-CoV-2 cell entry depends on ACE2 and TMPRSS2 and is blocked by a clinically proven protease inhibitor. Cell. 2020;181(2):271. 10.1016/J.CELL.2020.02.052. 32142651 10.1016/j.cell.2020.02.052 PMC7102627

[19] Fraunhofer-Gesellschaft. EBiSC - European Bank for Induced pluripotent stem cells. 2023. http://ebisc.org. Acessed 19 June 2023.

[20] D’Amour KA, Agulnick AD, Eliazer S, Kelly OG, Kroon E, Baetge EE. Efficient differentiation of human embryonic stem cells to definitive endoderm. Nat Biotechnol. 2005;23(12):1534–41. 10.1038/nbt1163. 16258519 10.1038/nbt1163

[21] Stabler CT, Morrisey EE. Developmental pathways in lung regeneration. Cell Tissue Res. 2017;367(3):677–85. 10.1007/s00441-016-2537-0. 27957616 10.1007/s00441-016-2537-0 PMC5321816

[22] Guillot L, Nathan N, Tabary O, Thouvenin G, Le Rouzic P, Corvol H, Amselem S, Clement A. Alveolar epithelial cells: master regulators of lung homeostasis. Int J Biochem Cell Biol. 2013;45:2568–73. 10.1016/J.BIOCEL.2013.08.009. 23988571 10.1016/j.biocel.2013.08.009

[23] Carcaterra M, Caruso C. Alveolar epithelial cell type II as main target of SARS-CoV-2 virus and COVID-19 development via NF-Kb pathway deregulation: a physio-pathological theory. Medical Hypotheses. 2021;146:110412. 10.1016/J.MEHY.2020.110412. 33308936 10.1016/j.mehy.2020.110412 PMC7681037

[24] Wong AP, Bear CE, Chin S, Pasceri P, Thompson TO, Huan LJ, Ratjen F, Ellis J, Rossant J. Directed differentiation of human pluripotent stem cells into mature airway epithelia expressing functional CFTR protein. Nat Biotechnol. 2012;30:876. 10.1038/nbt.2328. 22922672 10.1038/nbt.2328 PMC3994104

[25] Jackson CB, Farzan M, Chen B, Choe H. Mechanisms of SARS-CoV-2 entry into cells. Nat Rev Mol Cell Biol. 2022;23(1):3–20. 10.1038/S41580-021-00418-X. 34611326 10.1038/s41580-021-00418-x PMC8491763

[26] Bluhmki T, Traub S, Müller AK, Bitzer S, Schruf E, Bammert MT, Leist M, Gantner F, Garnett JP, Heilker R. Functional human iPSC-derived alveolar-like cells cultured in a miniaturized 96-transwell air–liquid interface model. Sci Rep. 2021;11(1):17028. 10.1038/S41598-021-96565-4. 34426605 10.1038/s41598-021-96565-4 PMC8382767

[27] Dye BR, Miller AJ, Spence JR. How to grow a lung: applying principles of developmental biology to generate lung lineages from human pluripotent stem cells. Curr Pathobiol Rep. 2016;4(2):47–57. 10.1007/s40139-016-0102-x. 27340610 10.1007/s40139-016-0102-x PMC4882378

[28] Nikolić MZ, Sun D, Rawlins EL. Human lung development: recent progress and new challenges. Development 2018;145(16):dev163485. 10.1242/dev.163485. 10.1242/dev.163485 PMC6124546 30111617

[29] Abo KM, de Aja JS, Lindstrom-Vautrin J, Alysandratos KD, Richards A, Garcia-De-Alba C, Huang J, Hix OT, Werder RB, Bullitt E, Hinds A, Falconer I, Villacorta-Martin C, Jaenisch R, Kim CF, Kotton DN, Wilson AA. Air-liquid interface culture promotes maturation and allows environmental exposure of pluripotent stem cell-derived alveolar epithelium. JCI Insight. 2022;7(6):e155589. 10.1172/JCI.INSIGHT.155589. 10.1172/jci.insight.155589 PMC8986076 35315362

[30] van Riet S, Ninaber DK, Mikkers HMM, Tetley TD, Jost CR, Mulder AA, Pasman T, Baptista D, Poot AA, Truckenmüller R, Mummery CL, Freund C, Rottier RJ, Hiemstra PS. In vitro modelling of alveolar repair at the air-liquid interface using alveolar epithelial cells derived from human induced pluripotent stem cells. Sci Rep. 2020;10(1):1–12. 10.1038/s41598-020-62226-1. 32218519 10.1038/s41598-020-62226-1 PMC7099095

[31] Schruf E, Schroeder V, Le HQ, Schönberger T, Raedel D, Stewart EL, Fundel-Clemens K, Bluhmki T, Weigle S, Schuler M, Thomas MJ, Heilker R, Webster MJ, Dass M, Frick M, Stierstorfer B, Quast K, Garnett JP. Recapitulating idiopathic pulmonary fibrosis related alveolar epithelial dysfunction in a human iPSC-derived air-liquid interface model. FASEB J 2020;34(6):7825–7846.10.1096/fj.201902926R 10.1096/fj.201902926R 32297676

[32] Huang J, Hume AJ, Abo KM, Werder RB, Villacorta-Martin C, Alysandratos KD, Beermann ML, Simone-Roach C, Lindstrom-Vautrin J, Olejnik J, Suder EL, Bullitt E, Hinds A, Sharma A, Bosmann M, Wang R, Hawkins F, Burks EJ, Saeed M, Wilson AA, Mühlberger E, Kotton DN. SARS-CoV-2 infection of pluripotent stem cell-derived human lung alveolar type 2 cells elicits a rapid epithelial-intrinsic inflammatory response. Cell Stem Cell. 2020;27(6):962. 10.1016/J.STEM.2020.09.013. 32979316 10.1016/j.stem.2020.09.013 PMC7500949

[33] Zhao Y, Zhao Z, Wang Y, Zhou Y, Ma Y, Zuo W. Single-cell RNA expression profiling of ACE2, the receptor of SARS-CoV-2. Am J Respir Crit Care Med. 2020;202(5):756–9. 10.1164/RCCM.202001-0179LE/SUPPL_FILE/DISCLOSURES.PDF. 32663409 10.1164/rccm.202001-0179LE PMC7462411

