# Supplementary material for: Alveolar epithelial-like cell differentiation in a dynamic bioreactor: a promising 3D-approach for the high-throughput generation of lung cell types from human induced pluripotent stem cells
Source: In Vitro Model. 2023 Jun 29;2(6):249–62. doi: 10.1007/s44164-023-00052-1 (PMC11756466; doi:10.1007/s44164-023-00052-1)
Supplement: Supplementary file 1 — Supplementary file1 (DOCX 2074 KB) [file 44164_2023_52_MOESM1_ESM.docx]

**Supplementary Material**

**Alveolar epithelial-like cell differentiation in a dynamic bioreactor: a promising 3D-approach for the high throughput generation of lung cell types from human induced pluripotent stem cells**

Michelle Müller^1^, Yvonne Kohl^1^, Anja Germann^1^, Sylvia Wagner^1^, Heiko Zimmermann^1,2,3*^, Hagen von Briesen^1^


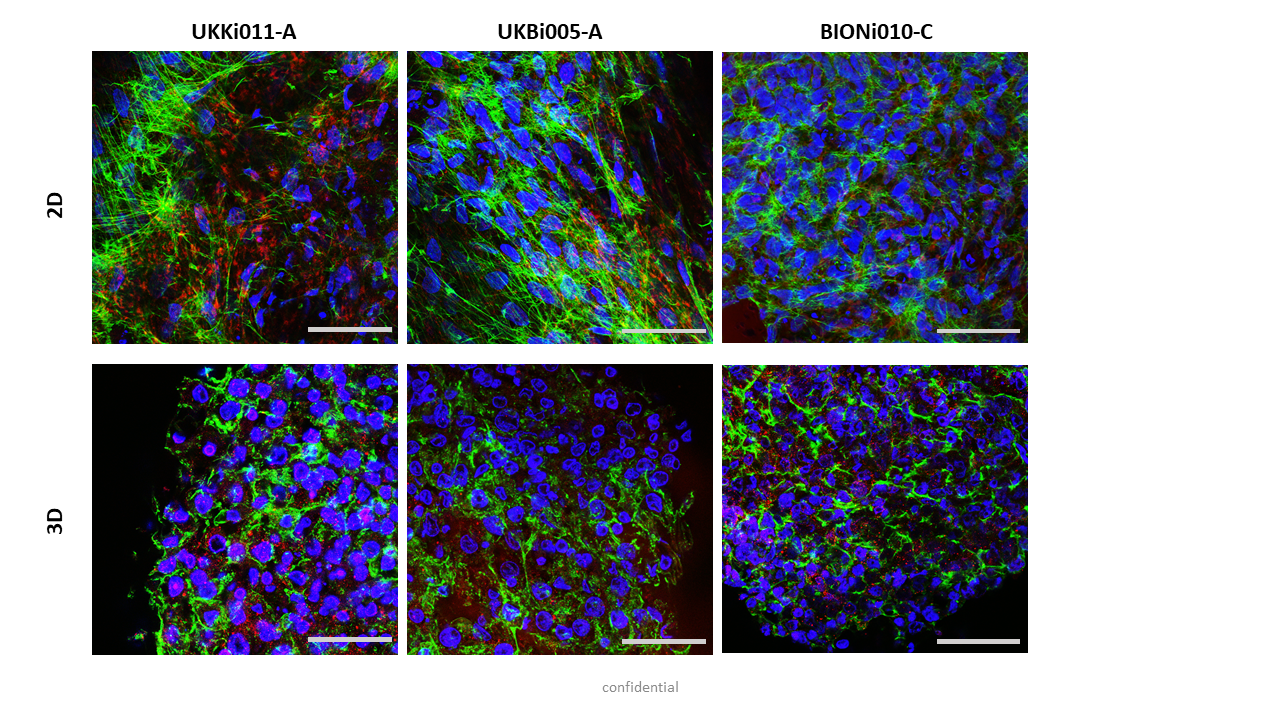


**Fig. S1:** Immuncytochemistry staining on day 30 of three hiPSC lines (UKKi011-A, UKBi005-A, BIONi010-C) from healthy donors differentiated in AEC2 under 2D- and 3D-conditions. Actin cytoskeleton (green), cell nuclei (blue) and SP-C (red). Scale bar 50 µm.


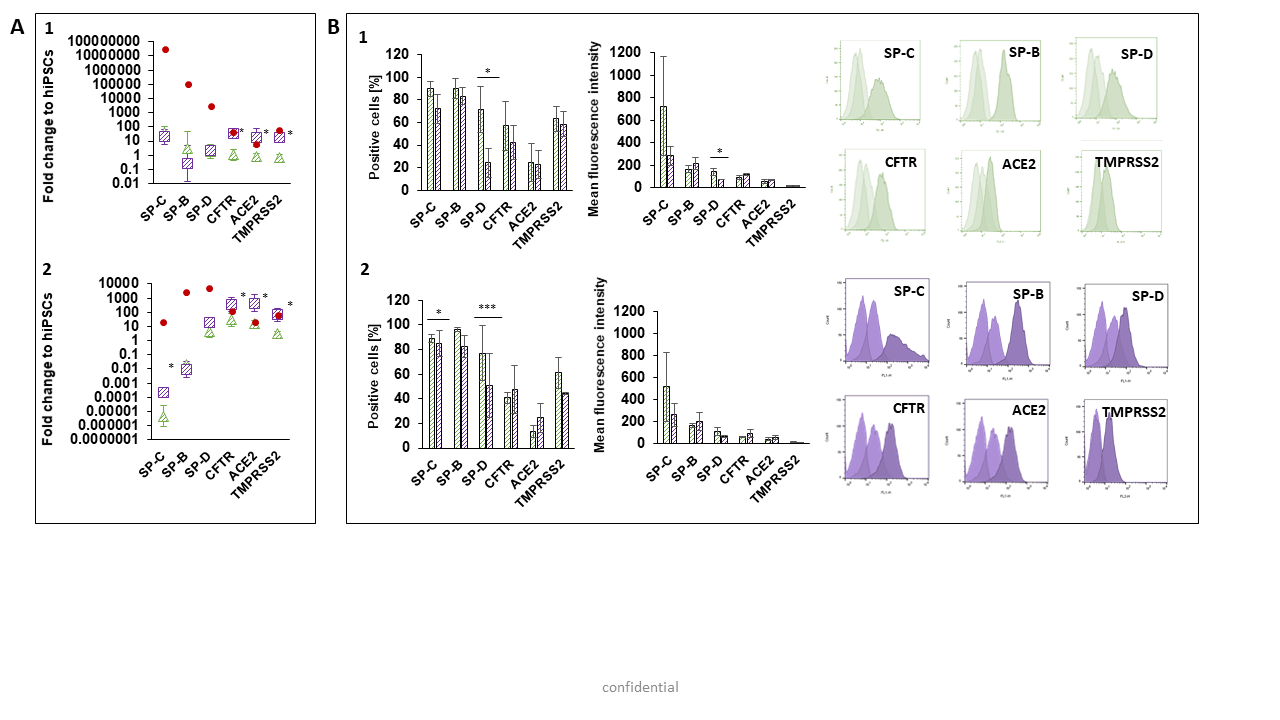


**Fig. S2:** **Analysis of Alveolar epithelial-like cells type 2 (AEC2) on day 23 differentiated from two hiPSC lines with CF mutation (F508del) under 2D (green) and 3D (purple) conditions.** **A**: Diagrams of gene expression show the fold change over undifferentiated hiPSCs of the gene of interest analysed by qPCR (2^-∆∆CT^ method). HPRT1 was used as endogenous reference gene. Red dots show gene expression of respective markers in adult human lung. **B:** Protein expression diagrams show the percentage of positive cells and the mean fluorescence intensity for the indicated markers analysed by flow cytometry (representative histograms of cell line 3 BIONi010‑C-O16). Data of three independent experiments (gene expression: mean ± CI 95 %; *p < 0.05; protein expression: mean ± SD, *p < 0.05, ***p < 0.001, unpaired two-tailed Student´s t-test). 1: BIONi010-C-O16; 2: DYP0250.


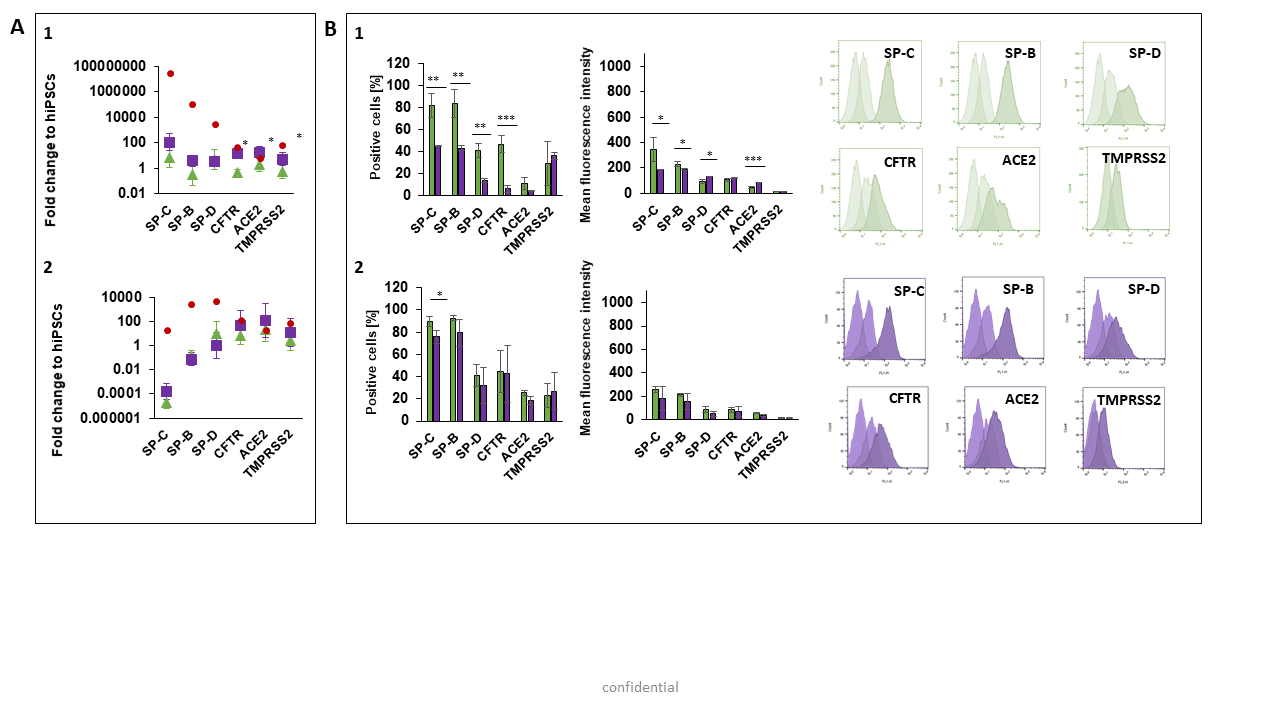


**Fig. S3:** **Analysis of Alveolar epithelial-like cells type 2 (AEC2) on day 30 differentiated from two hiPSC lines with CF mutation (F508del) under 2D (green) and 3D (purple) conditions.** **A**: Diagrams of gene expression show the fold change over undifferentiated hiPSCs of the gene of interest analysed by qPCR (2^-∆∆CT^ method). HPRT1 was used as endogenous reference gene. Red dots show gene expression of respective markers in adult human lung. **B:** Protein expression diagrams show the percentage of positive cells and the mean fluorescence intensity for the indicated markers analysed by flow cytometry (representative histograms of cell line 3 BIONi010‑C-O16). Data of three independent experiments (gene expression: mean ± CI 95 %; *p < 0.05; protein expression: mean ± SD, *p < 0.05, **p < 0.01, ***p < 0.001, unpaired two-tailed Student´s t-test). 1: BIONi010-C-O16; 2: DYP0250.

**Table S1: Antibodies**

| **Protein** | **Clone** | **Conjugate** | **Catalog No.** |
| --- | --- | --- | --- |
| ACE2 | Monoclonal Mouse IgG1 | AlexaFluor488 | Santa Cruz sc-390851 |
| AQP5 | Monoclonal Mouse IgG1 | AlexaFluor488 | Santa Cruz sc-514022 |
| CAV1 | Monoclonal Mouse IgG2B | AlexaFluor488 | Santa Cruz sc-53564 |
| CFTR | Monoclonal Mouse IgG1 | AlexaFluor488 | Santa Cruz sc-376683 |
| CXCR4 | Monoclonal Mouse IgG2A | AlexaFluor488 | R&D Systems FAB1701G-100G |
| FOXA2 | Polyclonal Goat IgG | AlexaFluor488 | R&D Systems IC2400G |
| NKX2.1 | Monoclonal Mouse IgG1 | AlexaFluor488 | Santa Cruz sc-53136 |
| PDPN | Monoclonal Mouse IgG2A | AlexaFluor488 | Santa Cruz sc-376695 |
| SP-B | Monoclonal Mouse IgG2B | AlexaFluor488 | Santa Cruz sc-133143 |
| SP-C | Monoclonal Mouse IgG2B | AlexaFluor488 | Santa Cruz sc-518029 |
| SP-D | Monoclonal Mouse IgG1 | AlexaFluor488 | Santa Cruz sc-25324 |
| SOX2 | Monoclonal Mouse IgG2A | AlexaFluor488 | R&D Systems IC2018G |
| SOX9 | Monoclonal Mouse IgG2A | AlexaFluor488 | Santa Cruz sc-166505 |
| SOX17 | Monoclonal Mouse IgG1 | AlexaFluor488 | R&D Systems IC19241G |
| TMPRSS2 | Monoclonal Mouse IgG1 | AlexaFluor594 | Santa Cruz sc-515727 |
| Isotype | Polyclonal Goat IgG | AlexaFluor488 | R&D Systems IC108G |
| Isotype | Monoclonal Mouse IgG1 | AlexaFluor488 | R&D Systems IC002G |
| Isotype | Monoclonal Mouse IgG2A | AlexaFluor488 | R&D Systems IC003G |
| Isotype | Monoclonal Mouse IgG2B | AlexaFluor488 | R&D Systems IC0041G |
| Isotype | Normal Mouse IgG1 | AlexaFluor488 | Santa Cruz sc-3890 |
| Isotype | Normal Mouse IgG2A | AlexaFluor488 | Santa Cruz sc-3891 |
| Isotype | Normal Mouse IgG2A | AlexaFluor594 | Santa Cruz sc-516620 |
| Isotype | Normal Mouse IgG2B | AlexaFluor488 | Santa Cruz sc-3892 |

**Table S2: Gene Expression Assays**

| **Gene Symbol** | **Assay ID** |
| --- | --- |
| ACE2 | Hs01085331_m1 |
| AQP5 | Hs00387048_m1 |
| CAV1 | Hs00971716_m1 |
| CFTR | Hs00357011_m1 |
| CXCR4 | Hs00607978_s1 |
| FOXA2 | Hs00232764_m1 |
| HPRT1 | Hs99999909_m1 |
| NKX2.1 | Hs00968940_m1 |
| PDPN | Hs00366766_m1 |
| SFTPB | Hs00167036_m1 |
| SFTPC | Hs00951326_g1 |
| SFTPD | Hs01108490_m1 |
| SOX2 | Hs00602736_s1 |
| SOX9 | Hs00165814_m1 |
| SOX17 | Hs00751752_s1 |
| TMPRSS2 | Hs05024838_m1 |
